# Case Report: Immune Microenvironment and Mutation Features in a Patient With Epstein–Barr Virus Positive Large B-Cell Lymphoma Secondary to Angioimmunoblastic T-Cell Lymphoma

**DOI:** 10.3389/fgene.2022.940513

**Published:** 2022-07-22

**Authors:** Fen Zhang, Wenyu Li, Qian Cui, Yu Chen, Yanhui Liu

**Affiliations:** Department of Pathology, Guangdong Provincial People’s Hospital, Guangdong Academy of Medical Sciences, Guangzhou, China

**Keywords:** angioimmunoblastic T-cell lymphoma, B-cell lymphoma, immune microenvironment, mutations, case report

## Abstract

On rare occasions, secondary Epstein–Barr virus (EBV)-associated B-cell lymphoma can develop in patients with angioimmunoblastic T-cell lymphoma (AITL). Here, we describe the tumor microenvironment and mutation features of a patient with EBV + large B-cell lymphoma (LBCL) secondary to AITL. He was admitted to hospital due to a 1-year history of fever and enlarged right inguinal lymph nodes. A biopsy of the right inguinal lymph node demonstrated that numerous diffuse medium-sized atypical lymphocytes proliferated, together with increased extrafollicular follicular dendritic cell meshwork, and the lymphocytes expressed CD3, CD4, BCL6, CD10, PD-1, CXCL13, and Ki-67 (75%). Thus, a diagnosis of AITL was made. However, the disease progressed following treatment by CHOP regimen (cyclophosphamide, adriamycin, vincristine, and prednisone). Biopsy showed that most of the cells were positive for CD20 staining and IgH rearrangement. Analysis of 22 kinds of immune cells showed that the numbers of activated NK cells and activated memory T cells increased, while the T-follicular helper population decreased in the transformed sample. In addition, compared with the primary sample, *RHOA (G17V)* mutation was not detected, while *JAK2* and *TRIP12* gene mutations were detected in the transformed sample. Overall, we described the immune microenvironment and mutation features of a patient with EBV + LBCL secondary to AITL. This study will help us to understand the mechanisms by which AITL transforms to B-cell lymphoma.

## Introduction

Angioimmunoblastic T-cell lymphoma (AITL) is a rare nodal peripheral T-cell lymphoma (PTCL), which is derived from T-follicular helper (TFH) cells and always accompanied by atypical medium-sized lymphocyte infiltrates (Attygalle et al., 2007; Swerdlow et al., 2016). In general, the scattering and proliferation of Epstein–Barr virus (EBV) + large B cells are commonly seen in AITL and even cause the transformation to EBV + diffuse large B-cell lymphoma (DLBCL) (Huang et al., 2012; Szablewski et al., 2019). Until now, only dozens of cases with EBV + DLBCL secondary to AITL have been reported (Zettl et al., 2002; Takahashi et al., 2010; Yang et al., 2012; Zhou et al., 2015; Chen et al., 2018; Lee et al., 2018), and further exploration of the transformed mechanisms is essential. Tumor microenvironment (TME) and mutation landscape are unique in different kinds of diseases and are closely associated with the prognosis of AITL and DLBCL (Keane et al., 2013; Xu-Monette et al., 2019; Pritchett et al., 2021; Yu et al., 2021). Here, we compared the immune cell populations and mutation features in one patient with AITL-transformed LBCL between the samples before and after transformation for the first time.

## Case Description

**FIGURE 1:**
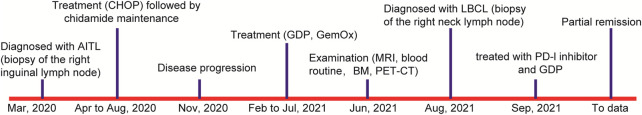
Important events of the patient according to the timeline.

**FIGURE 2:**
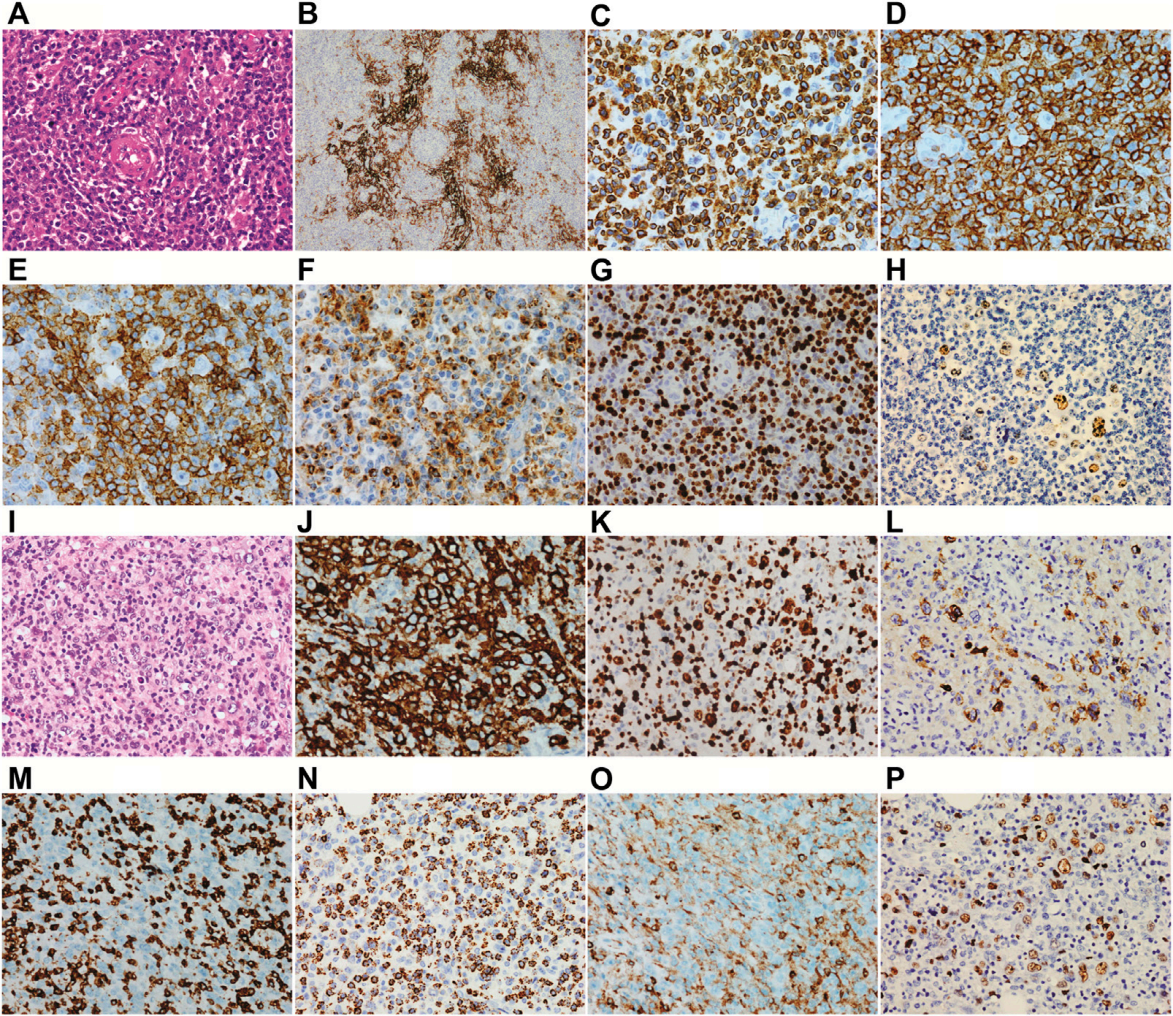
Histopathology of biopsy. **(A–H)** the first biopsy of the right inguinal lymph node which is diagnosed as angioimmunoblastic T-cell lymphoma and **(I–P)** the second biopsy of the right neck lymph node which is diagnosed as Epstein–Barr virus + diffuse large B-cell lymphoma. **(A)** a large number of diffuse medium-sized atypical lymphocytes proliferated with vascularity was observed. **(B)** increased extrafollicular follicular dendritic cell meshwork was observed. Immunohistochemical staining demonstrated that the lymphocytes were positive for CD3 **(C)**, CD4 **(D)**, PD-1 **(E)**, and CXCL13 **(F)**. **(G)** both the medium-sized T lymphocytes and scattered large B cells were positive for Ki67. **(H)** B cells were positive for EBER. Abnormal hyperplasia of large cells was observed **(I)**, and the cells were positive for CD20 **(J)**, Ki-67 **(K)**, and LMP1 **(L)** and negative for CD3 **(M)**. **(N)** the small lymphocytes were positive for TIA-1. **(O)** only the scattered small lymphocytes were positive for CD4, and the histiocytes were weakly positive for CD4. **(P)** most of the large B cells were positive for EBER.

In March 2020, a 56-year-old man was admitted to a hospital due to 1-year history of fever and enlarged right inguinal lymph nodes, with no family history. Figure 1 summarizes the important events of this patient according to the timeline. A cervicothoracic-enhanced computed tomography (CT) scan demonstrated multiple enlarged lymph nodes in the retroperitoneum, beside the right iliac artery and inguinal regions. A biopsy of the right inguinal lymph node demonstrated a large number of diffuse medium-sized atypical lymphocytes proliferated with vascularity (Figure 2A), together with increased extrafollicular follicular dendritic cell meshwork (Figure 2B). Immunohistochemical (IHC) staining demonstrated that the lymphocytes were positive for CD3 (Figure 2C), CD4 (Figure 2D), BCL6, CD10, PD-1 (Figure 2E), CXCL13 (Figure 2F), and Ki-67 (75%) (Figure 2G) staining. Moreover, obvious heteromorphic hyperplasia of large cells, including mononuclear cells similar to centroblasts, immunoblasts, Hodgkin cells, and Hodgkin/Reed–Sternberg-like cells, was observed, which presented scattered or a small focally aggregated distribution. These cells expressed CD20, PAX5, CD30, Ki-67, and EBV-encoded RNA (EBER), as detected by the IHC staining and *in situ* hybridization (Figure 2H). Capillary electrophoresis showed that the cells were negative for IgH and TCR gene rearrangements. A pathological diagnosis of AITL was made.

Then, the patient received a CHOP regimen (cyclophosphamide, adriamycin, vincristine, and prednisone) for seven cycles, followed by a maintenance therapy with chidamide, but the disease was partially progressed. Then, GDP (gemcitabine, dexamethasone, and cisplatin) and GemOx (gemcitabine and oxaliplatin) regimens were given. However, the curative effect was still dissatisfactory, with 1% lymphomatous cells in the bone marrow aspiration and multiple enlarged lymph nodes as detected by PET/CT. Next, a biopsy of the right neck lymph node was carried out. The normal structure of the lymph node was completely destroyed and the tumor tissues were mixed with a number of small lymphocytes and epithelioid histocytes and bits of plasma cells. To our surprise, TFH disappeared following 157 days of the first lymph node biopsy, together with abnormal hyperplasia of large cells (Figure 2I), which showed diffuse or focal distribution and were positive for CD20 (Figure 2J), CD79a, CD30, BCL6, BCL2, c-MYC, Ki-67 (Figure 2K), and LMP1 (Figure 2L) and negative for EBNA2 and CD3 (Figure 2M) staining. The small lymphocytes were positive for CD5, CD2, CD7, CD8, CD43, TIA-1 (Figure 2N), GrB, and Ki-67 (30%) and negative for CD10, PD-1, CXCL13, CD56, and CD30. The scattered small lymphocytes were positive for CD4, and histiocytes were weakly positive for CD4 (Figure 2O). In addition, the large B cells were positive for EBER (Figure 2P) and PD-L1 staining, as well as IgH gene rearrangement. Therefore, a diagnosis of EBV + DLBCL was made. Now the patient achieves partial remission following five cycles of PD-1 inhibitor and the GDP regimen and continues the treatment.

**FIGURE 3:**
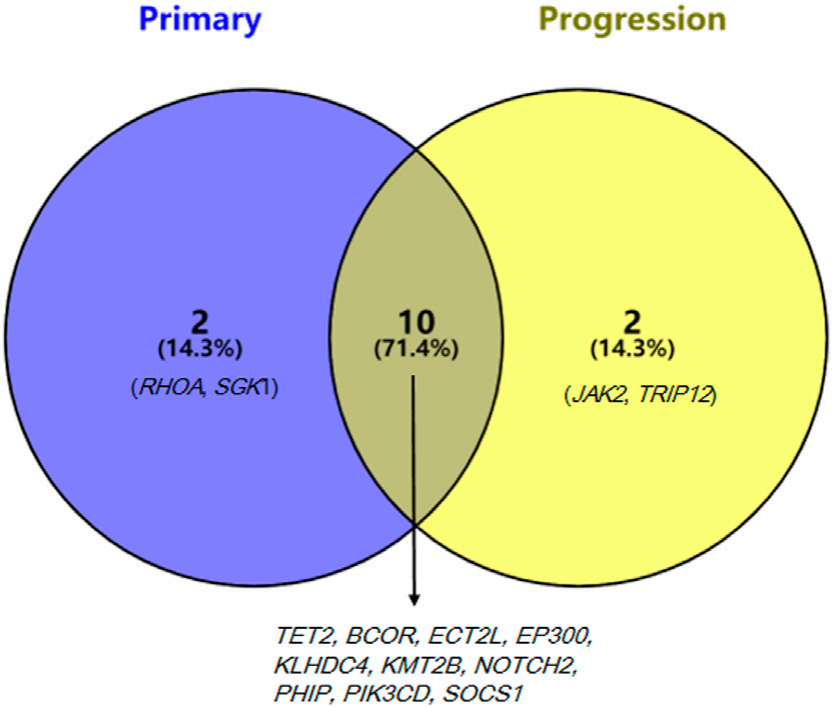
Mutated genes of the primary and progression samples.

Target-sequencing of the lymphoma-related 571 genes (Supplementary Table S1) was carried out to describe the mutation landscapes of the primary and transformed biopsy samples using Novaseq (Illumina, San Diego, CA, United States). The pathogenic mutations including the first level variations, which have been verified by large clinical trials and suggested by NCCN, ESMO guidelines, or the domestic and international expert consensus to present with clear clinical values in the diagnostic and treatment of lymphomas, and the second level variations that have certain values in the diagnostic and treatment of lymphomas verified by the small and medium-sized clinical trials were detected. The first level variations on *RHOA* and *TET2* genes and the second level variations on *BCOR*, *ECT2L*, *EP300*, *KLHDC4*, *KMT2B*, *NOTCH2*, *PHIP*, *PIK3CD*, *SGK1*, and *SOCS1* genes were identified in the primary sample, while the first level variations on *TET2* gene and the second level variations on *BCOR*, *ECT2L*, *EP300*, *JAK2*, *KLHDC4*, *KMT2B*, *NOTCH2*, *PHIP*, *PIK3CD*, *SOCS1*, and *TRIP12* genes were identified in the transformed sample. Mutations in 10 genes were detected in both primary and transformed samples, while *RHOA (G17V)* mutation disappeared and mutations in *JAK2* and *TRIP12* genes emerged in the transformed sample (Figure 3).

**FIGURE 4:**
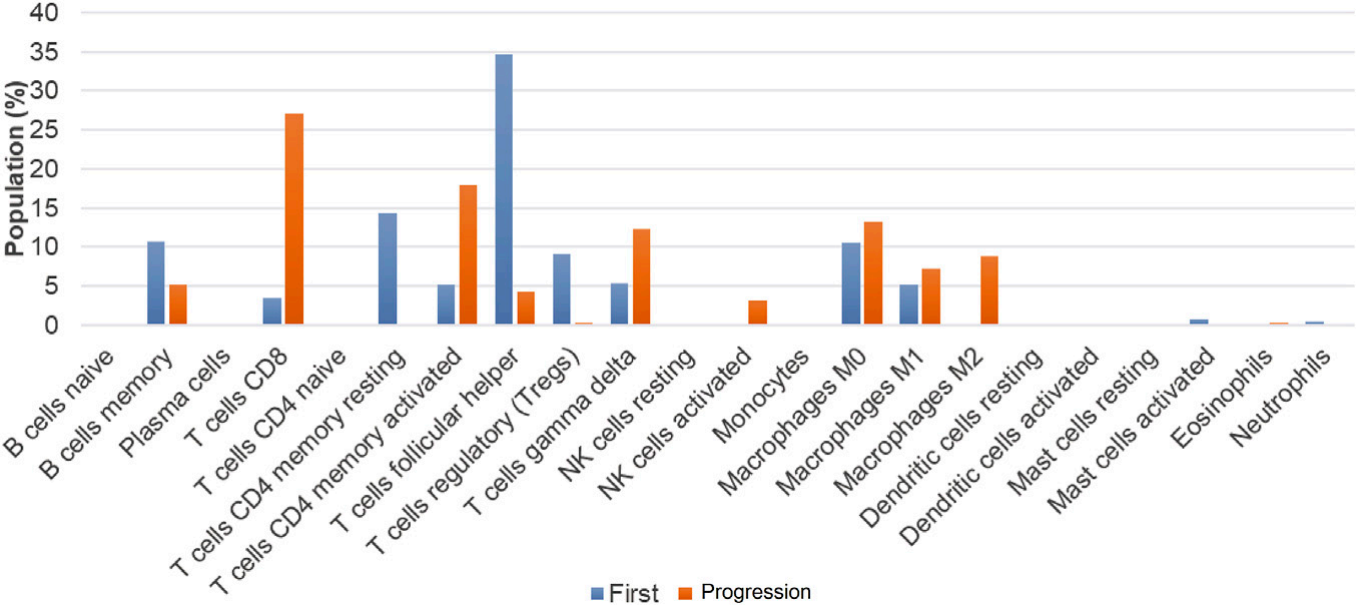
Differences of 22 types of immune cells between the primary and progression samples.

To further reveal the mechanism underlying the transformation from AITL to B-cell lymphoma, we performed the RNA-sequencing using Illumina Novaseq dual-end sequencing (2 × 150 bp) (Illumina, California, United States) to assess the TME. The count matrix of gene expression was assessed using Htseq-count (0.11.0). Then, 22 kinds of immune cell populations in the TME of the primary and transformed samples were compared by CIBERSORT deconvolution algorithm (v 1.03) as previously described (Yu et al., 2021). The numbers of memory B cells, TFH cells, regulatory T cells (Treg), and CD4^+^ T-memory resting cells were decreased in the transformed sample as compared with the primary sample, while the numbers of CD8+T cells, activated CD4^+^ memory T cells, γδT cells, activated NK cells, and M2 macrophages were increased (Figure 4).

## Discussion

Immune dysfunction and immunodeficiency are two main features of patients with AITL, which are often accompanied by EBV infection. EBV infection may further destroy the immune function of AITL patients, leading to B-cell dysfunction, and may trigger the occurrence of secondary B-cell lymphoma (Wang et al., 2021). In the current study, we report a rare case who developed LBCL following the treatment of AITL.

As per the previously reported cases (Zettl et al., 2002; Takahashi et al., 2010; Yang et al., 2012; Zhou et al., 2015; Chen et al., 2018; Lee et al., 2018), this patient carried EBV. We reasoned that the EBV protein signals (such as EBV-1 and LMP-1) may induce the secondary occurrence of B-cell lymphoma by promoting the proliferation of B cells (Dunleavy et al., 2007). Moreover, inconsistent lymphomas may originate from pluripotent stem cells that differentiate into B- and T-cell tumors due to genetic predisposition or prior exposure to specific therapies and mutagens (Wang et al., 2014). The EBV-negative DLBCL secondary to AITL was also reported, which might be AIDS (acquired immunodeficiency syndrome)-related lymphomas and caused by immunosuppressive status, chronic antigen stimulation, and cytokine deregulation (Knowles and Pirog, 2001; Shinohara et al., 2007).

The populations of memory B cells, TFH cells, Treg, and CD4 T-memory resting cells were decreased in the transformed LBCL sample as compared with the primary AITL sample, while the populations of CD8+T cells, activated CD4^+^ memory T cells, γδT cells, activated NK cells, and M2 were increased. In B-cell lymphoma, evidence has demonstrated that increases in the numbers of activated NK cells, memory T cells, and monocytes and decreases in the numbers of inactive NK cells, TFH, M0 macrophages, and activated mast cells were poor prognostic factors (Zhou et al., 2020; Yu et al., 2021). However, the role of tumor-associated macrophages (TAM) in the prognosis of DLBCL is controversial, which is related to the phenotypes of macrophages, M1 (CD68/HLA-DR), M2 (CD68/CD163), and therapeutic regimens. For instance, Riihijarvi et al. (Riihijarvi et al., 2015) found that CD68^+^ TAM and CD68 mRNA levels were associated with poor prognosis in DLBCL patients who received a CHOP regimen, while CD68^+^ TAM was associated with better prognosis in DLBCL patients treated with R-CHOP. Some researchers revealed that TAM-M2 was an adverse prognosis factor (Cioroianu et al., 2019), and also some studies showed that TAM was not significantly associated with patients prognosis (Cioroianu et al., 2019). At present, Treg association with the prognosis of DLBCL remains controversial (Chang et al., 2021). Taken together, we reason that the prognosis of this patient may be worse as the numbers of activated NK cells and activated memory T cells were increased, and the TFH cell number was decreased.

In addition, we assessed the mutation evolution profile of this patient. He carried *TET2* and *RHOA* (G17V) gene mutations at primary diagnosis, while *RHOA* (G17V) mutation disappeared and *JAK2* (c.1802_1804dupTGA, p. Met601dup) and *TRIP12* (c.1286_1288delGAA, p. Arg429del) mutations appeared in the transformed sample. *TET2, RHOA, DNMT3A*, and *IDH2* are the most common mutated genes identified in AITL, which affect approximately 80%, 50–70%, 20–40%, and 20–30% of the cases, respectively (Chiba and Sakata-Yanagimoto, 2020). However, mutations in *ARID1A*, *KMT2A*, *KMT2D*, *ANKRD11*, and *NOTCH2* genes are the most common variations in EBV + DLBCL patients, and the mutated genes were mainly enriched in NF-κB, IL6/JAK/STAT, and WT signaling pathways (Gebauer et al., 2021). RHOA belongs to the cell apoptosis/cell cycle signaling, and its mutation is a genetic hallmark of PTCL (Kataoka and Ogawa, 2016; Cortes et al., 2018). Expression of RHOA (G17V) in CD4^+^ T cells induced TFH cell specification and proliferation, and RHOA (G17V) expression together with TET2 loss resulted in the development of AITL in mice (Cortes et al., 2018). Zhou et al. (Zhou et al., 2019) found that *RHOA* was also a common mutated gene in EBV + DLBCL patients with a frequency of 33.3% (33.3%), and *RHOA* mutation has been demonstrated to be linked to a favorable prognosis in EBV + DLBCL patients. Thus, we speculate that this patient may have a bad prognosis due to the disappearance of *RHOA* mutation. *JAK2* is a member of the JAK family, which is related to aging, inflammation, hematopoiesis, and malignant transformation (Park et al., 2019). As mentioned above, mutations in EBV + DLBCL most popularly affect the JAK/STAT signaling, and we consider that JAK/STAT inhibitions may be attractive drugs for therapeutic intervention of EBV + DLBCL (Meyer and Levine, 2014). *TRIP12* (thyroid hormone receptor interacting protein 12), as a member of the structurally and functionally related E3 ubiquitin ligases, plays important roles in carcinogenesis through regulating the ubiquitination of key proteins (Brunet et al., 2020). Through describing the mutation profiles of this case, we conjecture *JAK2* and *TRIP12* mutations might be the indicators to predict the transformation of AITL to LBCL.

Several limitations should be clarified in the present study. We analyzed the immune microenvironment and mutation features in a patient with LBCL secondary to AITL for the first time, but only one sample was included. This is the main limitation of this study, and we intend to include more similar cases in future studies, as well as to reveal the roles of *JAK2* and *TRIP12* gene mutations in the transformation of AITL to LBCL *in vitro*.

Overall, we described the immune microenvironment and mutation evolution features in a rare case with LBCL secondary to AITL. It is crucial for further investigation with a larger number of cases to better understand the immune microenvironment and mutation features.

## Data Availability

The datasets for this article are not publicly available due to concerns regarding participant/patient anonymity. Requests to access the datasets should be directed to the corresponding author.
